# Case Report: staged segment-specific open and endovascular treatment of chronic post-dissection aortic disease with descending thoracic and giant infrarenal aneurysmal components

**DOI:** 10.3389/fcvm.2026.1925462

**Published:** 2026-10-12

**Authors:** Azat Chinaliyev, Mukhammejan Orazbay, Gulinur Chinaliyeva, Didar Khassenov, Tokan Sultanaliyev, Irlan Sagandykov, Aktolkyn Daumen

**Affiliations:** 1 Center of Interventional Radiology, National Research Oncology Center, Astana, Kazakhstan; 2 Center of Vascular and Reconstructive Surgery, National Research Oncology Center, Astana, Kazakhstan; 3 Doctoral School, Karaganda Medical University, Karaganda, Kazakhstan

**Keywords:** aorto-bi-iliac reconstruction, case report, chronic type B aortic dissection, descending thoracic aortic aneurysm, infrarenal aortic aneurysm, left subclavian artery, post-dissection aneurysm, TEVAR

## Abstract

**Background:**

Contemporary management of chronic post-dissection aortic disease includes open repair, thoracic endovascular aortic repair (TEVAR) with or without adjunctive false-lumen occlusion, fenestrated/branched endovascular repair, and staged open-endovascular strategies. Selection depends on anatomy, rupture risk, malperfusion, physiological reserve, and device availability.

**Case presentation:**

A 56-year-old woman with body mass index 47.2 kg/m^2^ and major cardiometabolic comorbidity presented with progressive abdominal pulsation. CTA demonstrated chronic Stanford type B/DeBakey III dissection with descending thoracic aneurysmal degeneration to 52.7 mm, a dominant symptomatic infrarenal aneurysmal component measuring approximately 11–12 cm, bilateral common iliac involvement, and residual paravisceral dissection without treatment-triggering enlargement or malperfusion. No Crawford extent was assigned because complete longitudinal centerline imaging was unavailable. Stage I comprised transperitoneal open aorto-bi-iliac reconstruction. After 118 days, elective morphology-driven zone 2 TEVAR was performed to cover the reported proximal entry region and reduce antegrade false-lumen inflow. Intentional left subclavian artery (LSA) coverage without prophylactic revascularization was followed by symptomatic subclavian steal requiring unplanned carotid-subclavian bypass.

**Results:**

No perioperative death, stroke, spinal cord ischemia, bowel ischemia, myocardial infarction, acute kidney injury, graft thrombosis, prosthetic infection, or access complication occurred. At latest follow-up, the patient was asymptomatic and all reconstructions were patent.

**Conclusion:**

This case illustrates competing priorities in segment-specific management of chronic post-dissection aortic disease. The unequivocal indication for urgent infrarenal repair contrasted with discretionary pre-emptive thoracic intervention below the conventional diameter threshold. The LSA-related complication emphasizes prophylactic LSA preservation/revascularization, spinal collateral planning, and lifelong surveillance of the untreated paravisceral segment after previous abdominal aortic reconstruction.

## Introduction

Current European Society for Vascular Surgery (ESVS) guidance emphasizes lifelong imaging surveillance and individualized intervention for chronic descending thoracic and thoracoabdominal aortic disease (1). Natural-history data from medically managed type B dissection identify aneurysmal degeneration as a major late reason for aortic intervention (2). Persistent false-lumen perfusion, distal re-entry flow, a rigid chronic septum, and branch vessels arising from different lumens may produce markedly different remodeling along the same aorta, so the indication for treatment can differ by segment.

Contemporary treatment options include conventional open replacement, TEVAR with or without adjunctive false-lumen treatment, fenestrated/branched endovascular aortic repair (F/BEVAR), and staged open-endovascular combinations (1, 3–6). Comparative evidence suggests that endovascular repair may reduce some early physiological burdens of extensive open repair but at the cost of greater reintervention, while spinal cord ischemia remains a concern with both strategies (3, 5). In chronic post-dissection anatomy, narrow true lumen, rigid septum, target-vessel incorporation, persistent distal false-lumen perfusion, and access-vessel anatomy can complicate endovascular planning (4, 6).

The educational value of this report does not lie in proposing a novel device or a universally preferable hybrid technique. Rather, it lies in transparent analysis of competing treatment priorities in heterogeneous chronic post-dissection disease: an unequivocal need to address a giant symptomatic infrarenal aneurysmal component, the discretionary nature of subsequent thoracic intervention below the usual diameter threshold, the consequences of elective LSA sacrifice, and the unresolved management of residual paravisceral false-lumen perfusion. The case is reported according to CARE guidance (7).

## Case description

A 56-year-old woman was referred with a visible and palpable abdominal pulsation that had progressed for approximately six months, beginning around November 2024. She was hemodynamically stable and had no clinical evidence of rupture, visceral malperfusion, limb ischemia, spinal cord ischemia, or focal neurological deficit. The exact date of the original acute type B dissection could not be reconstructed. No previous admission for a recognized acute dissection and no archived record documenting the initial decision for conservative management were found; therefore, the timing of the acute event and the reason for any prior non-operative management cannot be stated reliably. The first available diagnostic CT was dated 23 April 2025. The timeline of the episode of care is summarized in Table 1.

**Table 1:** Timeline of the episode of care.

Date/period	Event
November 2024	Onset of progressive visible and palpable abdominal pulsation.
23 April 2025	First available CT documented chronic Stanford type B/DeBakey III post-dissection aortic disease with descending thoracic aneurysmal dilatation, a dominant giant infrarenal aneurysmal component, and bilateral common iliac involvement.
30 May 2025	Planned Stage I: transperitoneal open infrarenal aneurysmotomy and 20 × 10 mm bifurcated aorto-bi-iliac prosthetic reconstruction.
5 June 2025	Discharged after Stage I with preserved bilateral lower-extremity perfusion.
July 2025	CTA confirmed aorto-bi-iliac graft patency and residual thoracic/paravisceral dissection.
25 September 2025	Planned Stage II: zone 2 TEVAR with two Valiant thoracic stent grafts and intentional LSA coverage.
27 November 2025	CTA after development of subclavian steal symptoms showed complete proximal LSA occlusion.
10 December 2025	Unplanned secondary left carotid-subclavian bypass using an 8-mm ringed graft.
March 2026	Latest documented follow-up: asymptomatic patient with patent thoracic, aorto-bi-iliac, and carotid-subclavian reconstructions.

Comorbidities included grade III arterial hypertension, hypertensive heart disease, coronary artery disease with stable angina, heart failure with preserved systolic function [New York Heart Association (NYHA) class I], dyslipidemia, type 2 diabetes mellitus, metabolic syndrome, subclinical hypothyroidism, and morbid obesity (BMI 47.2 kg/m^2^). No previous myocardial infarction, percutaneous coronary intervention (PCI), coronary stent, or coronary artery bypass grafting (CABG) was documented. Before admission, the recorded medication was perindopril/indapamide/amlodipine 5/2.5/10 mg once daily; no baseline antiplatelet, anticoagulant, statin, or glucose-lowering medication was documented. Glycated hemoglobin (HbA1c) was 6.69% on 3 December 2025.

Baseline serum creatinine was 64.3 μmol/L and eGFR was 92 mL/min/1.73 m^2^. Hemoglobin was 120 g/L, leukocyte count 6.01 × 10⁹/L, and platelet count 229 × 10⁹/L. INR was 0.94, activated partial thromboplastin time 24.5 s, and fibrinogen 3.7 g/L. Liver tests were normal, and spirometry showed no ventilatory impairment. Echocardiography was described as showing preserved left ventricular systolic function; a numeric ejection fraction and formal American Society of Anesthesiologists class were not recoverable from the archived record. No structured nutritional, prehabilitation, or pharmacological weight-loss program was documented before surgery. Prolonged postponement for weight reduction was not selected because the symptomatic infrarenal aneurysmal component measured approximately 11–12 cm and was considered to carry substantial rupture risk.

## Diagnostic assessment

The disease was characterized as chronic Stanford type B/DeBakey III post-dissection aortic disease with heterogeneous segment-specific aneurysmal degeneration. Preoperative CTA documented aneurysmal dilatation of the descending thoracic aorta, a dominant giant infrarenal aneurysmal component extending into both common iliac arteries, and an intervening chronically dissected paravisceral segment without documented malperfusion or segment-specific enlargement independently requiring renovisceral reconstruction.

On axial thoracic CTA, the maximum documented paired diameters were 52.7 × 43.5 mm. Sagittal reconstruction measured the descending thoracic aorta at 51.3 mm and showed the thoracic and giant infrarenal components within the same longitudinal field (Figure 1). At more distal lower descending/diaphragmatic levels, the aorta measured approximately 36–45 mm. The first available diagnostic CT was obtained on 23 April 2025; therefore, a reliable pretreatment thoracic growth rate could not be calculated.

**Figure 1:**
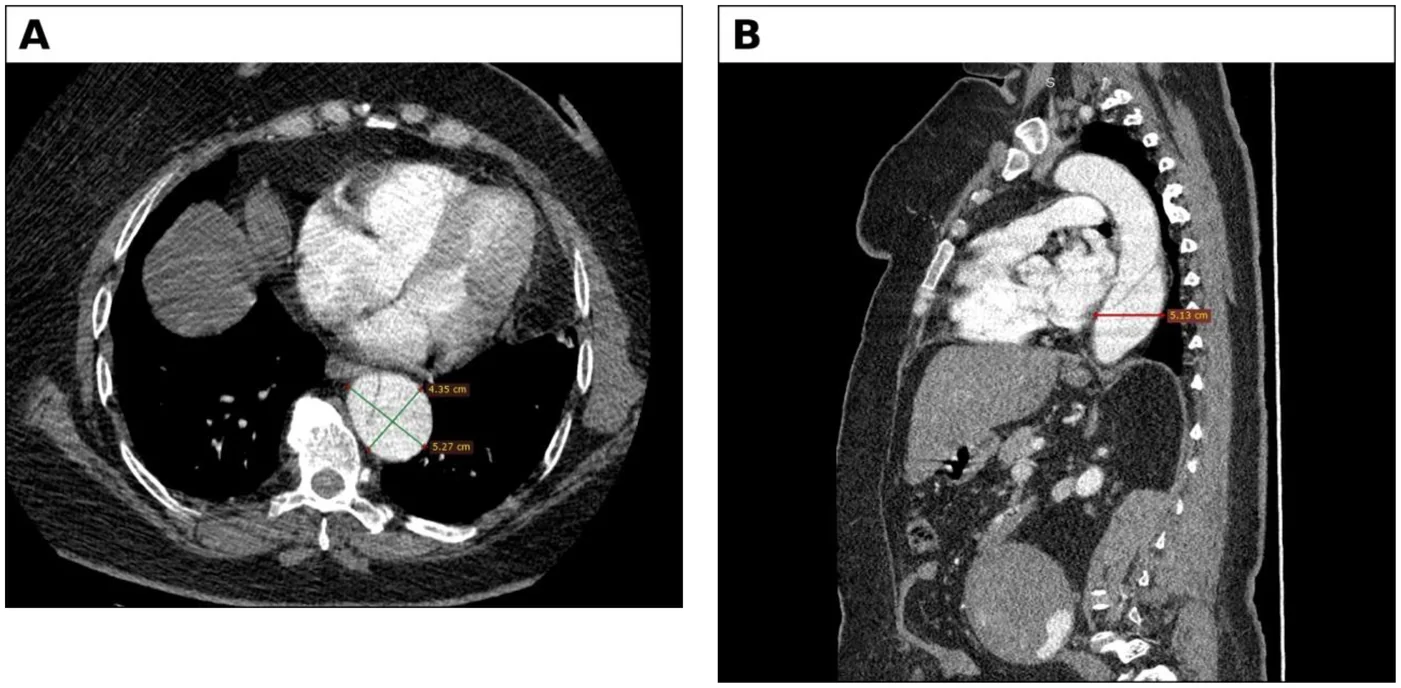
Preoperative CTA demonstrating the descending thoracic aneurysmal component and longitudinal post-dissection disease. **(A)** Axial thoracic CTA showing aneurysmal dilatation of the descending thoracic aorta with measured diameters of 52.7 × 43.5 mm. **(B)** Sagittal reconstruction showing a 51.3-mm descending thoracic diameter and the giant infrarenal aneurysmal component within the same longitudinal field. The intervening paravisceral aorta remained chronically dissected without documented treatment-triggering enlargement. The archived screenshots do not provide reliable centerline measurements of the LSA ostium or complete renovisceral branch anatomy.

The dominant infrarenal component measured 10.50 × 10.96 cm axially, 11.00 cm on coronal reconstruction, and 9.61 cm anteroposteriorly on sagittal reconstruction; its craniocaudal extent was approximately 12.61–13.37 cm. Intraoperative assessment showed an approximately 12-cm infrarenal aneurysm, a 15-mm infrarenal neck, marked infrarenal angulation of approximately 75 degrees, and bilateral common iliac aneurysmal involvement. Figure 2 includes the best archived axial, coronal, sagittal, and three-dimensional abdominal/aorto-iliac views available for this retrospective report. Exact common/external iliac and femoral access diameters were not recoverable.

**Figure 2:**
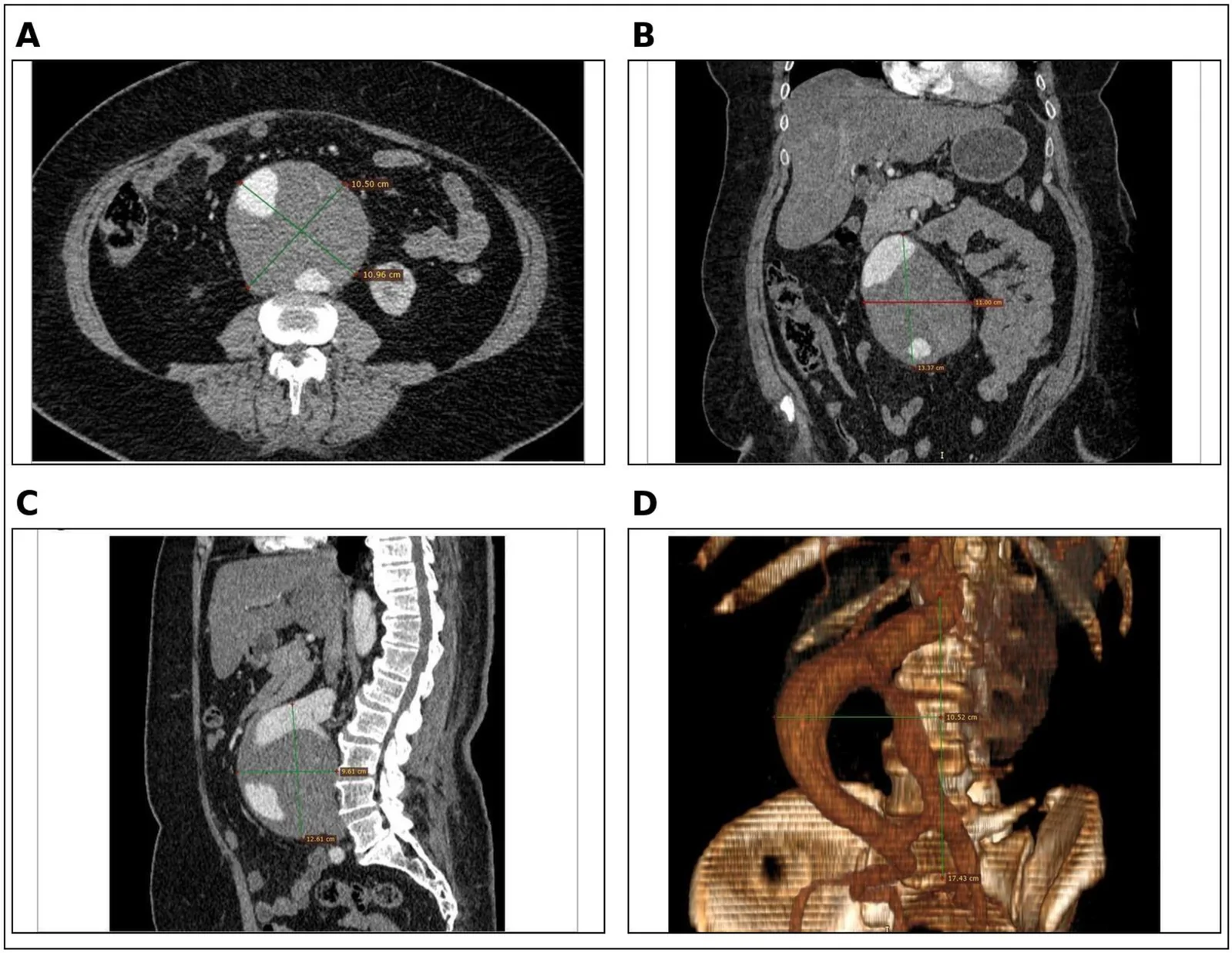
Available preoperative abdominal CTA and aorto-iliac reconstruction. **(A)** Axial image showing the 10.50 × 10.96 cm infrarenal aneurysmal component with false-lumen perfusion. **(B)** Coronal reconstruction showing a maximal transverse diameter of 11.00 cm and craniocaudal extent of 13.37 cm. **(C)** Sagittal reconstruction showing an AP diameter of 9.61 cm and craniocaudal extent of 12.61 cm; the adjacent upper abdominal/paravisceral aorta is included, although detailed branch-vessel centerline anatomy cannot be reconstructed from the archived dataset. **(D)** Enlarged three-dimensional reconstruction demonstrating marked infrarenal/aorto-iliac tortuosity and bilateral common iliac involvement.

The Crawford classification (extents I–V) is the widely used longitudinal framework for thoracoabdominal aortic aneurysms. However, the archived dataset does not contain a complete publication-quality arch-to-femoral arterial-phase centerline series, and the intervening paravisceral segment did not demonstrate comparable documented aneurysmal enlargement. A Crawford extent was therefore not assigned retrospectively. For TEVAR planning, the proximal landing zone was Ishimaru zone 2 and required intentional coverage of the LSA origin. The distal stent-graft terminated above the celiac artery; the exact distal extended Ishimaru zone, vertebral level, centerline seal length, and detailed entry/re-entry morphology were not recoverable. Likewise, formal pre-TEVAR documentation of vertebral dominance, circle-of-Willis adequacy, and LSA ostial dimensions was unavailable.

An extensive single-stage open thoracoabdominal replacement was considered excessively physiologically demanding. A totally endovascular strategy with F/BEVAR was considered technically feasible in a specialized high-volume program and was not rejected as anatomically impossible. It was not selected because the giant symptomatic 11–12 cm abdominal component required timely treatment, the infrarenal neck was short and angulated with bilateral iliac involvement, locally available off-the-shelf or physician-modified solutions were limited, and a custom-made fenestrated/branched device would have introduced a clinically relevant fabrication delay. Published real-world experience reports waiting periods approaching three months, with rupture during the waiting interval particularly in larger aneurysms (8). In this setting, reliance on a custom-made device was considered undesirable. The treatment plan therefore prioritized the dominant abdominal rupture risk, followed by elective thoracic intervention.

## Therapeutic intervention

### Stage I: open aorto-bi-iliac reconstruction

Stage I was performed on 30 May 2025 through a transperitoneal median laparotomy under general anesthesia. Exploration confirmed a giant approximately 12-cm chronic post-dissection infrarenal aneurysm with dense perianeurysmal adhesions and extension into both common iliac arteries. After 2,500 IU intravenous heparin, the infrarenal aorta and both common iliac arteries were cross-clamped. The proximal clamp was placed below the renal arteries, preserving suprarenal renal and visceral perfusion during the infrarenal reconstruction.

Aneurysmotomy exposed extensive mural thrombus and atherosclerotic debris. Bleeding lumbar arteries were individually suture-ligated. No surgical fenestration of the dissection flap was performed. Reconstruction used a 20 × 10 mm Albograft bifurcated prosthesis, with a proximal end-to-end anastomosis to the dissected infrarenal aorta and bilateral end-to-side anastomoses to the common iliac arteries. The operative report did not document felt/Teflon strip reinforcement of the proximal anastomosis or a deliberate maneuver to incorporate and fuse the true- and false-lumen layers; these techniques therefore cannot be claimed retrospectively.

The 363-minute operative duration reflected unusually difficult exposure associated with class III obesity, giant aneurysm size, dense perianeurysmal adhesions, bilateral iliac involvement, extensive thrombotic material, control of lumbar bleeding, and prolonged hemostasis. Estimated blood loss was 2,000 mL; 1,355 mL was reinfused using Cell Saver, and postoperative transfusion support was required. The prolonged duration should not be interpreted as representative of routine infrarenal aortic reconstruction. The patient remained in intensive care for four days and was discharged on postoperative day 6 with preserved bilateral lower-limb perfusion.

### Stage II: TEVAR

Stage II was performed on 25 September 2025, 118 days after Stage I. The indication was elective and morphology-driven rather than based on an established diameter or growth trigger. The descending thoracic aorta was chronically dissected and aneurysmally dilated to a maximum documented diameter of 52.7 mm. Because no earlier comparable CT was available, rapid growth could not be demonstrated. After the dominant abdominal rupture risk had been addressed, the procedural objective was to cover the reported proximal entry region, reduce antegrade false-lumen inflow and pressurization, and promote remodeling of the treated thoracic segment. This was therefore a pre-emptive intervention rather than repair mandated by conventional size or documented growth criteria.

Right common femoral surgical exposure was used for direct arterial control. The archived operative record does not preserve a specific patient-level reason for choosing open femoral cutdown rather than percutaneous large-bore access. Given the patient's severe obesity and the recognized groin-wound risk, percutaneous access would also have been a reasonable strategy if access anatomy and closure-device suitability permitted. This uncertainty is acknowledged rather than retrospectively reconstructed.

The proximal seal was placed in Ishimaru zone 2 with intentional coverage of the LSA origin. Two Valiant Thoracic stent grafts (Medtronic, Minneapolis, MN, USA), 34 × 167 mm proximally and 34 × 160 mm distally, were deployed under brief controlled hypotension of 70/40 mmHg. The distal component terminated immediately above the celiac origin. Long-segment coverage was selected to treat the dissected descending thoracic segment and cover the reported proximal entry region while preserving celiac perfusion; however, exact centerline landing-zone lengths, distal extended Ishimaru zone/vertebral level, planned oversizing, component overlap, and detailed distal re-entry anatomy were not recoverable from the archived planning worksheet. No distal false-lumen occluder was used.

Completion angiography showed satisfactory device position, no angiographically apparent type Ia, type Ib, or type III perigraft entry flow, and preserved celiac, superior mesenteric, and bilateral renal artery perfusion. Because the visceral aorta and distal re-entry communications remained untreated, persistent false-lumen perfusion was anticipated to remain possible and was not labeled as an endoleak. No prophylactic proximal LSA plug/coil embolization was documented, and the archived record does not preserve a specific rationale for omitting it. Available follow-up reports did not describe an LSA-related type II endoleak.

At the time of TEVAR, the LSA was intentionally covered without prophylactic revascularization because the patient had no baseline vertebrobasilar or left-arm ischemic symptoms and no previous CABG was documented. However, the archived record did not preserve a formal assessment of vertebral dominance, circle-of-Willis adequacy, or the contribution of LSA flow to the spinal collateral network. No prophylactic cerebrospinal fluid drain or motor-evoked/somatosensory-evoked potential monitoring was documented. Spinal cord risk mitigation relied on staging, preservation of renovisceral and pelvic perfusion, perioperative hemodynamic control, and serial neurological examinations. The patient was discharged on postoperative day 4 without neurological, renal, or access-site complication.

### Secondary left carotid-subclavian revascularization

Approximately six weeks after TEVAR, the patient developed progressive dizziness, headache, left-arm fatigue, and intermittent digital numbness. Blood pressure was 130/80 mmHg in the right arm and 80/60 mmHg in the left arm; the right radial pulse was palpable and the left was absent. CTA on 27 November 2025 showed complete proximal LSA occlusion. The archived preoperative and pre-bypass source records used for this report do not document a high-grade left common carotid stenosis; accordingly, no such lesion is attributed to the patient in this manuscript.

On 10 December 2025, an unplanned secondary left carotid-subclavian bypass was performed through a left supraclavicular approach. An 8-mm ringed prosthetic graft was tunneled beneath the sternocleidomastoid muscle and anastomosed end-to-side to the left common carotid and left subclavian arteries. No carotid endarterectomy was performed. Antegrade subclavian flow and distal pulsation were restored. Blood loss was approximately 100 mL, and no perioperative neurological event occurred.

## Follow-up and outcomes

There was no perioperative mortality during the complete treatment course. No stroke, transient ischemic attack, spinal cord ischemia, bowel ischemia, myocardial infarction, acute kidney injury, graft thrombosis, prosthetic infection, or access-site complication was recorded. Nevertheless, symptomatic subclavian steal after intentional LSA coverage constituted a clinically meaningful treatment-related complication and required an unplanned third vascular procedure.

The latest documented outpatient assessment occurred in March 2026, approximately three months after the bypass; the exact day and contemporaneous creatinine/eGFR values were not recoverable from the archived outpatient record. The patient was ambulatory and free of chest, back, abdominal, vertebrobasilar, or upper-extremity ischemic symptoms. Blood pressure was clinically controlled, neurological examination showed no focal deficit, the left radial pulse was restored, and upper- and lower-extremity perfusion was satisfactory.

Follow-up CTA showed patent thoracic endografts, aorto-bi-iliac prosthesis, and carotid-subclavian bypass, without migration, graft thrombosis, or reported type I/III perigraft entry flow (Figure 3). No interval aneurysm progression was reported. Because the paravisceral segment and distal re-entry communications were not sealed, complete thrombosis of the entire residual false lumen was not claimed. Medication at follow-up included aspirin 100 mg daily, clopidogrel 75 mg daily to complete six months, atorvastatin 10 mg daily, and antihypertensive therapy; no anticoagulant or glucose-lowering drug was documented.

**Figure 3:**
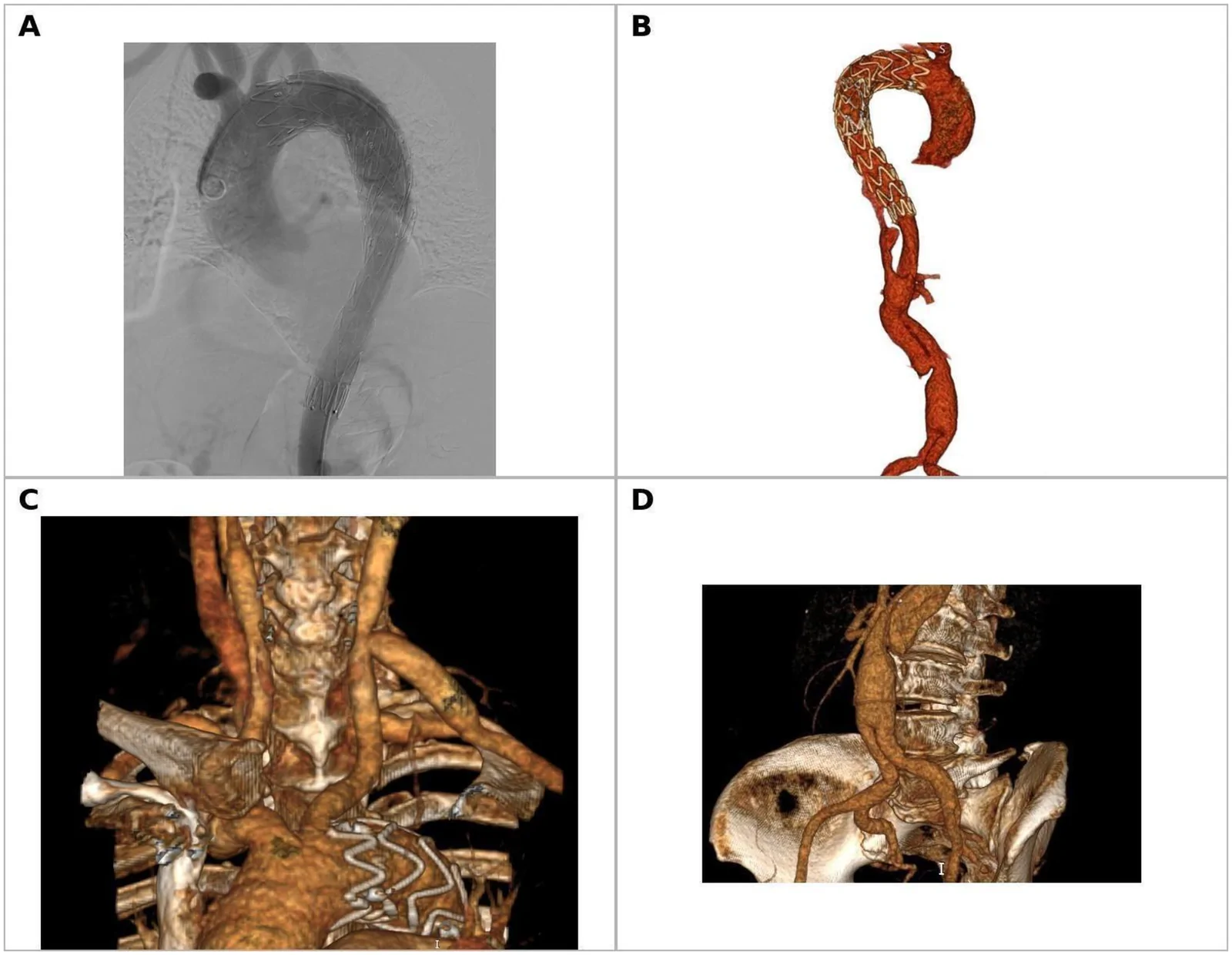
Endovascular repair and early follow-up. **(A)** Completion angiography after zone 2 TEVAR demonstrating the thoracic endograft construct with intentional coverage of the LSA origin. **(B)** Follow-up three-dimensional reconstruction of the thoracic endografts; the residual untreated paravisceral dissected segment lies distal to the construct. **(C)** Patent left carotid-subclavian bypass. **(D)** Patent bifurcated aorto-bi-iliac prosthesis.

## Discussion

The approximately 11-12 cm symptomatic infrarenal component was far beyond usual elective treatment thresholds and provided an unequivocal indication for Stage I. A previous report from our center described individualized open reconstruction for a complicated infected infrarenal aneurysm, illustrating the need to adapt repair to the clinical context (9). The descending thoracic aorta also demonstrated aneurysmal degeneration to 52.7 mm, whereas the intervening paravisceral segment remained chronically dissected without treatment-triggering enlargement or malperfusion. The anatomy therefore represented heterogeneous segment-specific post-dissection disease rather than a uniformly aneurysmal thoracoabdominal segment. Open and complex endovascular strategies carry different trade-offs: contemporary comparative evidence suggests similar early mortality in selected cohorts, lower renal injury and permanent spinal cord injury with endovascular repair in some analyses, but higher reintervention and important durability considerations (3).

A totally endovascular F/BEVAR strategy was technically conceivable in an experienced aortic center and should not be described as anatomically contraindicated. In this patient, however, the dominant symptomatic 11–12 cm abdominal component required timely treatment, whereas a custom-made fenestrated/branched device could have introduced a fabrication delay. Published experience has documented waiting intervals of approximately 12 weeks and rupture during the waiting period, particularly in patients with larger aneurysms (8). Therefore, in the setting of urgent rupture concern, reliance on a custom-made device was considered undesirable. Off-the-shelf branched or physician-modified solutions might have shortened this interval but were not available in the treating program (6).

A reasonable alternative sequence would have been thoracic-first repair with mandatory LSA preservation/revascularization, consideration of proximal LSA occlusion when required to prevent retrograde branch-related perigraft flow, and adjunctive distal false-lumen occlusion to promote thoracic false-lumen thrombosis. After short-interval inpatient monitoring, abdominal reconstruction could then have been performed after approximately 7–10 days. At the open abdominal stage, felt/Teflon reinforcement of the dissected proximal neck to incorporate the dissected layers could also have been considered. This alternative is clinically credible and is now acknowledged explicitly. The abdominal-first sequence used in this case was selected because the giant symptomatic infrarenal component was judged to represent the most immediate rupture threat. Staged strategies may reduce spinal cord risk, but they also expose patients to interval aortic events (5).

The abdominal-first strategy also carried a specific hazard: aortic cross-clamping can increase afterload and proximal arterial pressure, potentially stressing the untreated thoracic segment. No dedicated pre-positioned thoracic endovascular bailout device or other specific rupture bailout strategy was documented during Stage I. This is an important limitation of the chosen sequence. A thoracic-first strategy with LSA preservation and early abdominal completion would therefore also have been reasonable.

The indication for Stage II must be distinguished from the unequivocal indication for Stage I. The maximum documented thoracic diameter of 52.7 mm established aneurysmal degeneration, but current ESVS guidance retains 60 mm as the principal diameter threshold for elective intervention in chronic type B dissection (1). Rapid growth could not be demonstrated because 23 April 2025 was the first available CT. Repair of the infrarenal component alone followed by thoracic imaging surveillance was therefore a reasonable alternative. Stage II should be understood as an individualized morphology-driven, pre-emptive intervention, and this case does not establish that such TEVAR was necessary or superior to surveillance (1, 4).

Standard TEVAR cannot ensure complete false-lumen thrombosis when distal re-entry channels remain open. In this patient, the visceral/paravisceral segment was not sealed, so persistent retrograde false-lumen perfusion remained possible despite proximal thoracic entry coverage. Adjunctive techniques such as distal false-lumen plugs, Candy-Plug, Knickerbocker, coils, or other occlusion strategies can be considered when persistent false-lumen perfusion contributes to aneurysmal growth (10). A distal false-lumen occluder at the level of the distal thoracic component would have been a reasonable alternative intended to promote thoracic false-lumen thrombosis.

LSA management is the major treatment-planning limitation and educational point of this case. ESVS guidance recommends considering LSA revascularization when elective TEVAR requires intentional LSA coverage, particularly when extensive aortic coverage or previous open/endovascular aorto-iliac surgery threatens the spinal collateral network (1). Both factors applied after Stage I. In retrospect, absence of baseline arm or vertebrobasilar symptoms was insufficient justification for elective LSA sacrifice in this high-risk collateral setting, and prophylactic carotid-subclavian bypass or transposition should have been favored.

Dedicated off-the-shelf arch branch systems, including the Gore TAG Thoracic Branch Endoprosthesis and other single-branch platforms, can preserve antegrade LSA flow in suitable anatomy (11). Such devices were not available locally, but surgical LSA revascularization was available. No prophylactic proximal LSA embolization was documented; if a preserved or bypassed LSA were associated with retrograde perigraft perfusion, branch occlusion could be considered according to anatomy. The subsequent symptomatic subclavian steal requiring an unplanned bypass should be regarded as a preventable treatment-related complication and a learning point rather than support for selective delayed revascularization.

Spinal cord protection in extensive thoracic and thoracoabdominal aortic intervention is multimodal. Relevant measures include staging, preservation or revascularization of the LSA when appropriate, maintenance of pelvic/hypogastric and renovisceral collateral perfusion, avoidance of perioperative hypotension, maintenance of adequate oxygen delivery and hemoglobin, selective cerebrospinal fluid drainage, and neurological or MEP/SSEP monitoring in selected high-risk patients (1, 5, 12). In this patient, staging and collateral preservation were used, but prophylactic CSF drainage, MEP/SSEP monitoring, and a numeric postoperative mean arterial pressure target were not documented. No spinal cord ischemia occurred; this favorable neurological outcome should not be interpreted as validation of the original LSA strategy.

Severe obesity increased the risks of laparotomy, respiratory complications, wound morbidity, and femoral exposure. No structured nutritional, prehabilitation, or pharmacological weight-loss program was documented. Prolonged delay for weight reduction was considered undesirable because of the symptomatic giant infrarenal component. Similarly, the archived record does not preserve a patient-specific reason for surgical femoral cutdown at Stage II; percutaneous access could have reduced groin-wound risk if anatomy and closure-device suitability were favorable. Female sex was not considered a contraindication to F/BEVAR, although sex-specific evidence supports careful assessment of access-vessel caliber, tortuosity, and perioperative risk (13).

The untreated paravisceral dissection remains a site of potential future enlargement and requires lifelong surveillance. CTA should be performed at approximately 1 and 12 months after TEVAR and annually if stable; once dimensional stability is established, alternating non-contrast CT and CTA may be considered when device integrity and false-lumen perfusion remain assessable, and annual MRI/MRA is a reasonable radiation- and contrast-sparing alternative (1). Future paravisceral enlargement could require F/BEVAR, adjunctive false-lumen occlusion, or open reconstruction. The principal limitations of this report are its single-patient design, short follow-up, unplanned secondary LSA bypass, prolonged 118-day interstage interval, absence of serial pretreatment CT for thoracic growth assessment, incomplete archived centerline/branch-vessel planning data, and inability to assign a defensible Crawford extent. Its clinical value is therefore not proof of superiority or novelty of the operative strategy, but a transparent account of competing segment-specific priorities, reasonable alternative strategies, and the consequences of incomplete LSA protection.

## Patient perspective

Before treatment, the patient reported increasing anxiety caused by the visible abdominal pulsation and fear of rupture. She stated that the staged explanations helped her understand why different segments required different procedures. After carotid-subclavian bypass, dizziness and arm fatigue resolved. At the latest visit she was ambulatory, free of vascular symptoms, and satisfied with her functional recovery.

## Conclusion

Management of this patient required segment-specific decisions rather than treatment of a single uniformly aneurysmal thoracoabdominal segment. Open aorto-bi-iliac reconstruction addressed the unequivocally indicated giant symptomatic infrarenal component. Subsequent zone 2 TEVAR represented an elective morphology-driven, pre-emptive intervention for a 52.7-mm descending thoracic post-dissection aneurysmal segment; infrarenal repair alone with surveillance would also have been reasonable. Intentional LSA coverage without prophylactic revascularization resulted in symptomatic subclavian steal and an unplanned carotid-subclavian bypass. The principal lessons are the importance of LSA preservation and spinal collateral planning during extensive elective thoracic coverage, the need to consider persistent distal false-lumen perfusion when the visceral segment remains untreated, and lifelong imaging surveillance of the residual paravisceral dissection.

## Data Availability

All relevant anonymized clinical data are included in this article. Additional anonymized information may be made available by the corresponding author on reasonable request and in accordance with institutional policy.
